# Establishment of a Drug Screening Model for Cardiac Complications of Acute Renal Failure

**DOI:** 10.3390/biom11091370

**Published:** 2021-09-16

**Authors:** Shuyi Liao, Wenmin Yang, Ting Yu, Lu Dai, Xiaoliang Liu, Jiangping Zhang, Jinghong Zhao, Chi Liu

**Affiliations:** 1 Department of Nephrology, The Key Laboratory for the Prevention and Treatment of Chronic Kidney Disease of Chongqing, Chongqing Clinical Research Center of Kidney and Urology Diseases, Xinqiao Hospital, Army Medical University (Third Military Medical University), Chongqing 400037, China; mutanlsy@gmail.com (S.L.); yangwm0901@163.com (W.Y.); dailu923@163.com (L.D.); liusnliang@163.com (X.L.); zhjp2009@163.com (J.Z.); 2 Department of Respiratory Medicine, Xinqiao Hospital, Army Medical University (Third Military Medical University), Chongqing 400037, China; doct3yuting@163.com

**Keywords:** acute renal failure (ARF), cardiorenal syndrome (CRS), zebrafish model, drug screening, nitroreductase (NTR), metronidazole (MTZ)

## Abstract

Acute renal failure (ARF) is a clinical critical syndrome with rapid and severe decline of renal function. Complications of ARF, especially its cardiac complications (cardiorenal syndrome type 3, CRS-3), are the main causes of death in patients with ARF. However, the shortage and limited efficacy of therapeutic drugs make it significant to establish new large-scale drug screening models. Based on the Nitroreductase/Metronidazole (NTR/MTZ) cell ablation system, we constructed a *Tg(cdh17:Dendra2-NTR)* transgenic zebrafish line, which can specifically ablate renal tubular epithelial cells. The absence of renal tubular epithelial cells can lead to ARF in zebrafish larvae. The ARF symptoms, such as heart enlargement, slow heart rate and blood stasis, are similar to the clinical manifestations of human CRS-3. Furthermore, two therapeutic drugs (digoxin and enalapril) commonly used in the clinical treatment of heart failure were also effective in alleviating the symptoms of CRS-3 in zebrafish, which proved the effectiveness of this model. Drug screening further discovered a potential drug candidate, α-lipoic acid, which can effectively alleviate the symptoms of CRS-3 through its antioxidant function. Accordingly, we established a new ARF model of zebrafish, which laid a foundation for large-scale screening of new therapeutic drugs for its complications.

## 1. Introduction

Acute renal failure (ARF) is the most severe stage of acute renal injury (AKI). When ARF occurs, the decrease of the glomerular filtration rate (GFR), inflammation surge and renal endocrine dysfunction will lead to complications in the cardiovascular system, blood system and nervous system [1]. Among complications, cardiac complications (cardiorenal syndrome type 3, CRS-3) are the main causes of death in patients with ARF. Presently, the treatment of CRS-3 is mainly for acute heart failure and hypertension. The therapeutic drugs used are cardiotonic glycosides (positive inotropic drugs, such as digoxin) and non-cardiotonic glycosides (the angiotensin-converting enzyme inhibitor (ACEI), such as enalapril). However, these drugs have obvious side effects, such as digitalis poisoning during digoxin treatment, which lead to the aggravation of renal dysfunction and hypotension [2]. Therefore, it is important to develop safer and more effective drugs.

The zebrafish, a vertebrate model organism, is widely used to study developmental mechanisms and disease pathologies. Studies show 84% of human genes associated with the disease have a counterpart in zebrafish [3,4]. Zebrafish have the characteristics of abundant strains, small size and rapid embryo development in vitro. Regarding the advantages of experimental technology, it possesses optical transparency, easy genetic manipulation and complete annotation of the genome sequence. Especially as a model of kidney disease, zebrafish have multiple advantages, such as simple kidney structure, high similarity with human kidney disease, as well as the ability to repair and regenerate after injury [5]. Zebrafish are also used for high-throughput in vivo drug screening due to their fast reproduction and high yield. Therefore, zebrafish can be used to establish a large-scale drug screening model for ARF complications.

Bacterial nitroreductase (NTR) can catalyze the reduction of the nontoxic prodrug metronidazole (MTZ) to produce cytotoxic products (a cross-linking agent between DNA) that can induce cell death [6]. Based on the NTR/MTZ system, we can construct transgenic fish that express the fluorescent protein-NTR fusion protein in specific tissues through tissue-specific promoters. Then, the transgenic zebrafish can be exposed to a specific concentration of the MTZ solution, which would cause acute tissue-specific ablation [7]. A previous study shows that the MTZ/NTR system can specifically damage NTR-expressing cells without damaging the surrounding cells [8]. The NTR/MTZ system has been successfully utilized to induce conditional targeting of a variety of cell ablations for regeneration studies in zebrafish, such as renal podocytes [9,10,11], cerebrovascular cells [12], liver cells [13] and pancreatic beta cells [8]. As the NTR/MTZ system can easily obtain a large number of individuals with specific tissue damage at the same time, the research of tissue injury repair and regeneration is greatly accelerated. However, the model of renal tubule specific ablation has not been constructed yet.

Herein, in order to obtain zebrafish ARF models with complete acute renal tubular ablation, we generated the *Tg(cdh17: Dendra2-NTR)* transgenic zebrafish line. Upon exposure to MTZ, transgenic zebrafish larvae showed symptoms similar to human ARF, such as edema, heart enlargement, slow heart rate and blood stasis. The cardiac complications are highly similar to the clinical manifestations of human CRS-3. Subsequently, we treated ARF zebrafish larvae with two commonly used cardiac drugs, digoxin and enalapril. The positive effects observed verified the applicability of drug screening with the zebrafish larvae ARF model. Finally, we tried to use this model to screen potential therapeutic drugs. Among these drugs, α-Lipoic acid can significantly improve the symptoms of CRS-3 through its activity of antioxidant stress. In general, we constructed a new zebrafish ARF model, which lays a foundation for further screening of potential therapeutic drugs for the treatment of clinical CRS-3 or other ARF complications.

## 2. Materials and Methods

### 2.1. Zebrafish Maintenance

According to the zebrafish book, all adult zebrafish (*Danio rerio*) are raised in aquaculture facilities with temperature control and standard light cycles (14 h of light, 10 h of darkness). The zebrafish larvae are fed in egg water at 28 °C (deionized water containing 0.2% Instant Ocean Salt; pH 6.9–7.2; electrical conductivity 480–510 μs/cm). Since the zebrafish larvae are nourished by the yolk sac, all 7 dpf (post-fertilization) larvae in this study did not need to be fed. The following zebrafish lines were used: AB strain, *Tg(cdh17:Dendra2-NTR)*, *Tg(cmlc2:GFP)* and *Tg(flk1:GFP;gatal:DsRed)*. All experimental procedures of zebrafish are carried out in accordance with the internationally recognized principles of experimental animal use and care ethics.

### 2.2. Generation of Tg(cdh17-Dendra2-NTR) Transgenic Line

The promoter of *cdh17*, a 4265 bp DNA fragment, was amplified from zebrafish genomic DNA using specific primers (FORWARD) 5′-GAACAAAAGCTGGGTAC CGGGCCCAGAAAGTGCTGATAGTCCCAAG-3′; (REVERSE) 5′-CATGGTGGC GACCGGTGGATCCCCTCGTCCCAAGCAGTAAAACCTGATG-3′. Then, we cloned the promoter of cdh17, the fluorescent protein gene *Dendra2* and the *NTR* gene sequence into the pBluescript II vector. After obtaining *cdh17*:*Dendra2*-*NTR* constructs, the constructs were injected into zebrafish embryos of the AB genetic background at the one-cell stage for transgenesis [14].

### 2.3. NTR/MTZ Induced Renal Tubular Epithelial Cells Ablation

Zebrafish *Tg(cdh17-Dendra2-NTR)* larvae were collected at about 72 hpf for subsequent experiments. Metronidazole (MTZ) (M3761-100G, Merck, Shanghai, China) was freshly prepared in egg water containing 0.2% dimethyl sulfoxide (DMSO) (D8418, Merck, Shanghai, China). The larval fish were treated with various concentrations (2 mM, 4 mM, 6 mM, 8 mM, 10 mM) of MTZ for 8, 16, 20 and 24 h in the dark. Subsequently, they were washed with fresh egg water at least 3 times, then zebrafish larvae were returned to 28 °C and monitored every 6 to 12 h. The minimum number of larvae was 30 per group under each treatment condition, and each experiment was repeated more than three times.

### 2.4. FITC-Inulin Clearance Assay

Five percent (*w*/*v*) FITC-Inulin (I121221, Aladdin, Shanghai, China) was dissolved into 0.9% NaCl and dialyzed with a 1000 Da dialysis bag to remove any unbound FITC. Anesthetized larvae were injected with 2 nL of the FITC-inulin solution via the heart. Fifteen minutes post-injection, the FITC intensity of larvae placed on 1% agar was determined by imaging over the caudal region. Subsequently, larvae were immediately transferred to 70 μL egg water. After 4 h, the FITC intensity of larvae was reimaged [15]. ImageJ (Version 1.53j, National Institutes of Health: USA, 2021) software was used to calculate intensity values for each larva.

### 2.5. Determination of Uric Acid and Urea Nitrogen

Thirty zebrafish larvae of *Tg(cdh17:Dendra2-NTR)* were collected at 1 dpi to 4 dpi, washed with ultrapure water and placed in 1 mL ultra-pure water. Consequently, the supernatant was obtained by low-temperature centrifugation after homogenization on ice. The uric acid and urea nitrogen from the supernatant were detected by the Urea Nitrogen Assay Kit, Micromethod (D799850, Sangon, Shanghai, China) and the Uric Acid (UA) Content Assay Kit, Micromethod (D799286, Sangon, Shanghai, China) according to the manufacturer’s instructions.

### 2.6. Apoptosis Detection

*Tg(cdh17:Dendra2-NTR;cmlc2:GFP)* larvae treated with MTZ and control were washed three times using fresh egg water and cultured in 28 °C egg water; subsequently, they were collected at 1 dpi to 4 dpi, then fixed with 4% paraformaldehyde at 4 °C overnight. The TUNEL Assay (C10617, Thermo Fisher Scientific, Shanghai, China) was used to detect the cell apoptosis of the kidney and heart according to the manufacturer’s instructions.

### 2.7. Drug Treatment

Immediately after MTZ elution, ARF larvae of (*cdh17*:*Dendra2*-*NTR*;*cmlc2*:*GFP*) were randomly divided into an experimental group and a control group for therapeutic drug treatment. The experimental group was respectively treated with two clinical cardiac drugs, digoxin (NSC95100, Selleck, Shanghai, China) (0.1 μg/mL, 0.5 μg/mL) and enalapril (MK-422, Selleck, Shanghai, China) (25 μg/mL, 50 μg/mL), as well as three potentially effective drugs, α-lipoic acid (A506197, Sangon, Shanghai, China) (4 μM, 8 μM), curcumin (A600346, Sangon, Shanghai, China) (0.5μM, 1 μM) and cinnamaldehyde (A501968, Sangon, Shanghai, China) (2 μg/mL, 4 μg/mL). Each drug was diluted with egg water containing 0.2% DMSO, then treated for 2 h a day for 4 days. Simultaneously, the control group was treated with 0.2% DMSO egg water.

### 2.8. ROS Detection

Reactive oxygen species (ROS) were determined using the Reactive Oxygen Species Assay Kit (S0033S, Beyotime, Shanghai, China) based on 2′,7′-dichlorodihydrofluorescein diacetate (DCFH-DA). The ARF zebrafish larvae, treated or untreated with α-lipoic acid, were incubated with 10 mM DCFH-DA (diluted with egg water containing 0.2% DMSO) in the dark at 28 °C for 1 h. Then, they were washed three times with fresh egg water. The fluorescence intensity was monitored using a stereo-fluorescence microscope with excitation at 488 nm.

### 2.9. Acquisition Phenotype Data

In order to continuously observe the ARF phenotype and post-injury cardiac complications, 3–5 larvae were randomly selected from each treatment condition, then their body, heart, blood vessels and erythrocyte phenotypes were recorded every day for 4 consecutive days. The larvae were placed in 1% low-melting-point agarose SFR (TM) High Resolution (A600234, BBI Life Sciences, Shanghai, China). The heartbeat and erythrocytes’ movement videos were collected by a stereoscopic fluorescence microscope, the BX3-CBH microscope (Olympus, Japan), and the morphological changes of blood vessels (dorsal aorta (DA) and posterior cardinal vein (PCV), and vessels around the heart) were photographed by the Nikon A1 confocal microscope. After fixation with 4% formaldehyde for 1 h, the heart morphology of MTZ-treated or untreated larvae was photographed by the Nikon A1 confocal microscope.

### 2.10. Assessment of Heart Rate and Blood Flow

The heart rate of zebrafish larvae was calculated according to the method in reference [16]. Firstly, videos were processed using Adobe Premiere CC software, VirtualDub software, X264vfw software and ImageJ software. ImageJ was used to analyze the dynamic pixel changes. Secondly, the peak analyzer function in Origin 9.1 was used to calculate the peak time. Finally, the peak time interval and the number of beats per minute (BPM) were calculated using Microsoft Excel software based on the peak time. The smooth heartbeat rhythm graph was done by Origin 9.1 software with default settings.

The frame-by-frame motion of erythrocytes from the video image was tracked using ImageJ to estimate the tail artery blood flow velocity in the posterior aorta [17,18]. More than four erythrocytes of each fish (5 zebrafish larvae in each group) over 10 frames at a video framerate of 30 frames/second were analyzed to determine the average erythrocyte velocity (mm/seconds).

## 3. Results

### 3.1. Generation of Tg(cdh17:Dendra2-NTR) Transgenic Line

**Figure 1:**
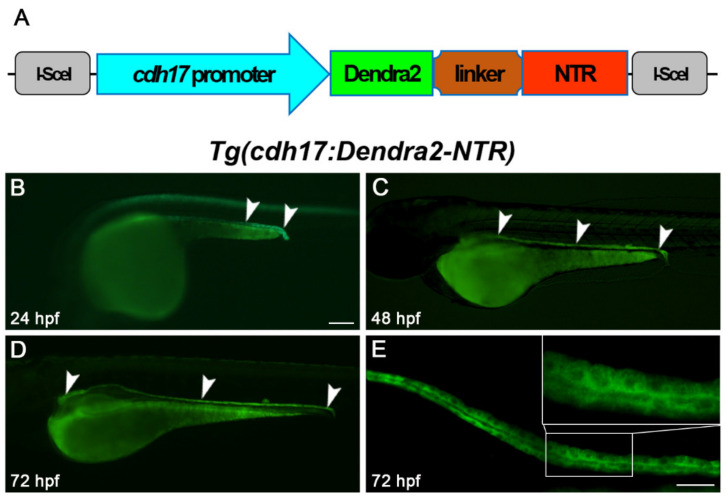
*Tg(cdh17:Dendra2-NTR)* transgenic line construction. (**A**) Constructs used to generate the *cdh17*-driven *Dendra2*-*NTR* transgenic line is shown. Dendra2, green-to-red photo-switchable fluorescent protein; NTR, nitroreductase. Dendra2 expression in *Tg(cdh17:Dendra2-NTR)* transgenic fish renal tubules (arrowheads) at 24 hpf (**B**), 48 hpf (**C**) and 72 hpf (**D**), respectively. Scare bar: 200 μm. (**E**) Detailed display of the laser confocal image in the middle section of the 72 hpf *Tg(cdh17:Dendra2-NTR)* transgenic fish renal tubule. Scare bar: 100 μm.

We amplified a 4.265 kb DNA fragment located upstream of the *cdh17* promoter. Subsequently, *Dendra2*-*NTR* was ligated under the control of this promoter in the pBluescript II vector, and zebrafish embryos were injected with these constructs (Figure 1A). By crossing with wild-type fish, we identified founders of *Tg(cdh17*:*Dendra2*-*NTR*) transgenic fish lines. The founders showed that Dendra2 was expressed exclusively in the region of renal tubules at 24, 48 and 72 h post-fertilization (hpf) by fluorescence microscopy (Figure 1B–E). The founder with the highest expression of Dendra2 was utilized to collect embryos for further research and line maintenance. At the same time, we found that the *Tg(cdh17*:*Dendra2*-*NTR*) transgenic line can completely mark the proximal and distal tubule of the pronephron. We also did not find a high expression of Dendra2 in other types of cells. Therefore, this transgenic line can be used for subsequent ablation of renal tubular epithelial cells.

### 3.2. Establishment of Zebrafish Larvae ARF Model

**Figure 2:**
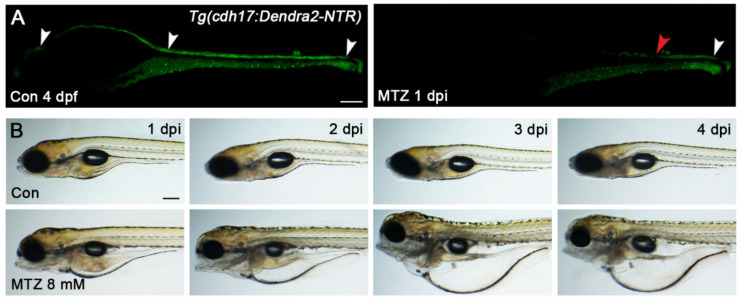
MTZ induces renal tubular epithelial cells-specific ablation. (**A**) Treatment with MTZ resulted in the attenuation of the Dedra2 signal in the renal tubules of *Tg(cdh17:Dendra2-NTR)* zebrafish larvae (*n* = 30). The white arrowheads in Con larvae indicated the intact renal tubules (head, body and tail). The white and red arrowhead in MTZ larvae respectively indicated the remaining and the injured parts of the renal tubules. Scare bar: 100 μm. (**B**) Edema could be seen in MTZ-treated *Tg(cdh17:Dendra2-NTR)* zebrafish larvae but not in the control at 1 dpi to 4 dpi (*n* = 30). Scale bar: 200 μm. Con, control. n, the number of larvae per group under each treatment condition. Each experiment was repeated more than three times.

Next, we explored the conditions MTZ-induced renal tubular epithelial cells’ ablation. As the renal tubules and many important organs of zebrafish larvae have been developed at 72 hpf, we chose this period to construct the ARF model. The *Tg(cdh17:Dendra2-NTR)* zebrafish larvae (Heterozygotes of *Tg(cdh17:dendra2-NTR)* were used in all of the following experiments) at 72 hpf were treated with MTZ for 8, 12, 16, 20 and 24 h at concentrations ranging from 2 to 10 mM. There are only two situations after MTZ treatment: One is the large-scale loss of renal tubular epithelial cells, in which the renal tubules cannot be repaired. The other is that all cells will not be ablated. A partially repairable tubular injury was not found. When treated with low-concentration MTZ, the renal tubule of most zebrafish larvae will not be ablated, but the proportion of renal-tubule-ablated larvae begins to rise after increasing the concentration (Figure S1). When the zebrafish larvae were treated with 8 mM MTZ for 16 h, the ideal effect was found whereby 100% of zebrafish larvae showed a significant loss of Dedra2 fluorescence in the renal tubules (Figure 2A). Furthermore, edema occurred in the larvae after treatment with 8 mM MTZ (Figure 2B), similar to human ARF. Although the same effect could also be observed under 10 mM MTZ treatment for 12 h or less than 8 mM MTZ for more than 16 h, we selected the condition of 8 mM MTZ treatment for 16 h as a default method since the non-specific toxicity may be caused by the higher concentration or longer duration of the MTZ treatment. In order to verify whether MTZ is toxic to renal function, we treated the 72 hpf *Tg(cdh17:DsRed)* larvae with 8 mM MTZ for 16 h. The renal function was not affected, and the apoptosis of renal tubular epithelial cells did not increase, which proved that 8 mM MTZ would not cause toxicity to zebrafish kidneys (Figure S2).

### 3.3. Renal Tubules Injury Mediated the Renal Function Loss

**Figure 3:**
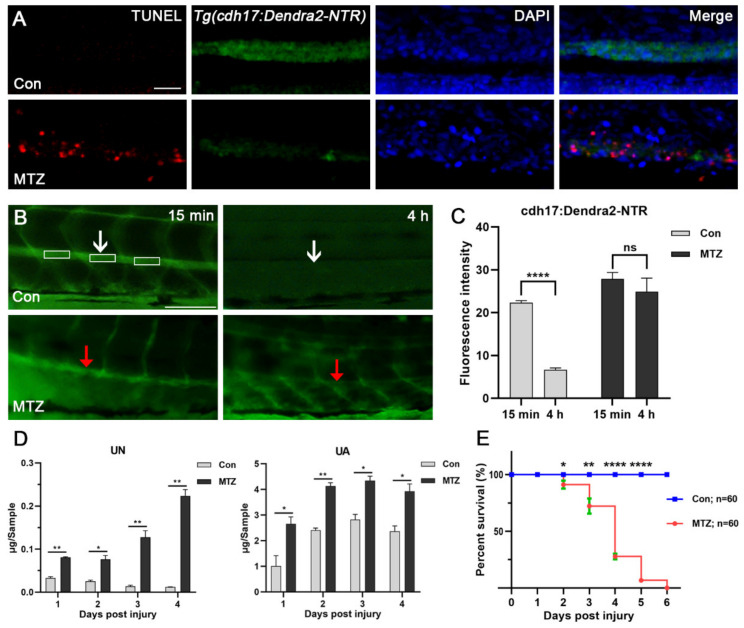
Renal tubular injury leads to loss of renal function. (**A**) Terminal deoxynucleotidyl transferase-mediated deoxyuridine triphosphate nick end labeling (TUNEL)-positive apoptotic cells (red) were detected in the renal tubules of *Tg(cdh17:Dendra2-NTR)* zebrafish larvae treated with MTZ, but not in the control (*n* = 30). Scale bar: 50 μm. (**B**,**C**) *Tg(cdh17:Dendra2-NTR)* zebrafish larvae treated with MTZ or the control were injected with 2 nL 5% FITC-inulin, and FITC-inulin fluorescence intensity of the caudal artery was detected in three separate regions 15 min and 4 h after injection. White or red arrows respectively indicated the same measuring position of Con larvae or MTZ larvae. (**D**) Zebrafish larvae treated with MTZ or control zebrafish larvae (30 larvae/Sample) were collected at 1 dpi to 4 dpi for the detection of UA and UN. (**E**) *Tg(cdh17:Dendra2-NTR)* zebrafish larvae treated with MTZ and control zebrafish larvae were collected (*n* = 60, respectively), and their survival rate was calculated from 1 dpi to 6 dpi. The data are expressed as the mean ± SEM; * *p* < 0.05, ** *p* < 0.01, and **** *p* < 0.001 (ANOVA/Dunnett’s test).

NTR-expressing cells could convert non-toxic MTZ into cytotoxic substances through NTR to induce cell death [6]. We further investigated whether MTZ can induce apoptosis of renal tubular epithelial cells by TUNEL assay. As a result, we found obvious apoptosis occurred in the renal tubules of MTZ-treated *Tg(cdh17:Dendra2-NTR)* larvae compared with the control (Figure 3A). These results suggested that the loss of Dendra2 fluorescence in *Tg(cdh17:Dendra2-NTR)* larvae were caused by the apoptosis of renal tubular epithelial cells. Inulin is a non-metabolic polysaccharide that can be filtered freely but will not be reabsorbed or secreted by the nephron. The clearance rate of inulin is an important criterion for the detection of the glomerular filtration rate (GFR) [15]. Thus, we used FITC-inulin to evaluate the renal function of zebrafish in the ARF model. Our results showed that the FITC-inulin removal degree in ARF fish was significantly reduced compared with the control group, which confirmed the loss of renal function in the zebrafish ARF model (Figure 3B,C). Uremic toxins are always accumulated inside the body if they have not been cleared in a timely manner after ARF occurs. Uremic toxin is a driving factor of CRS-3, and is often related to uremic cardiomyopathy and heart rhythm disorders [19]. Urea nitrogen (UN) and uric acid (UA) have been widely used as essential clinical markers for the diagnosis and monitoring of renal function in patients with nephropathy [19]. In order to evaluate the effect of renal tubular epithelial cells’ ablation on renal function, we detected the changes of these urotoxins in MTZ-treated *Tg(cdh17*:*Dendra2-NTR)* larvae and the control. Thirty ARF or control larvae were gathered to determine UN and UA from 1-day post injury (dpi) to 4 dpi. After treatment with MTZ, the average levels of UN and UA of the larvae were increased about 2 times compared with the control (Figure 3D). This is similar to the accumulation of urotoxins in human ARF patients without intervention. In order to evaluate the survival of zebrafish larvae after ARF, we calculated the survival rate of ARF larvae. Larvae were placed in a six-well plate, with each well containing 15 fish. After MTZ treatment, the number of deaths was counted every day until all the larvae died. ARF larvae died from 2 dpi. Average survival rates from 2 dpi to 5 dpi were 91.11%, 72.22%, 27.78% and 6.667%, respectively (Figure 3E). No larvae survived at 6 dpi.

### 3.4. Zebrafish ARF Model Can Simulate Human CRS-3 Complications

**Figure 4:**
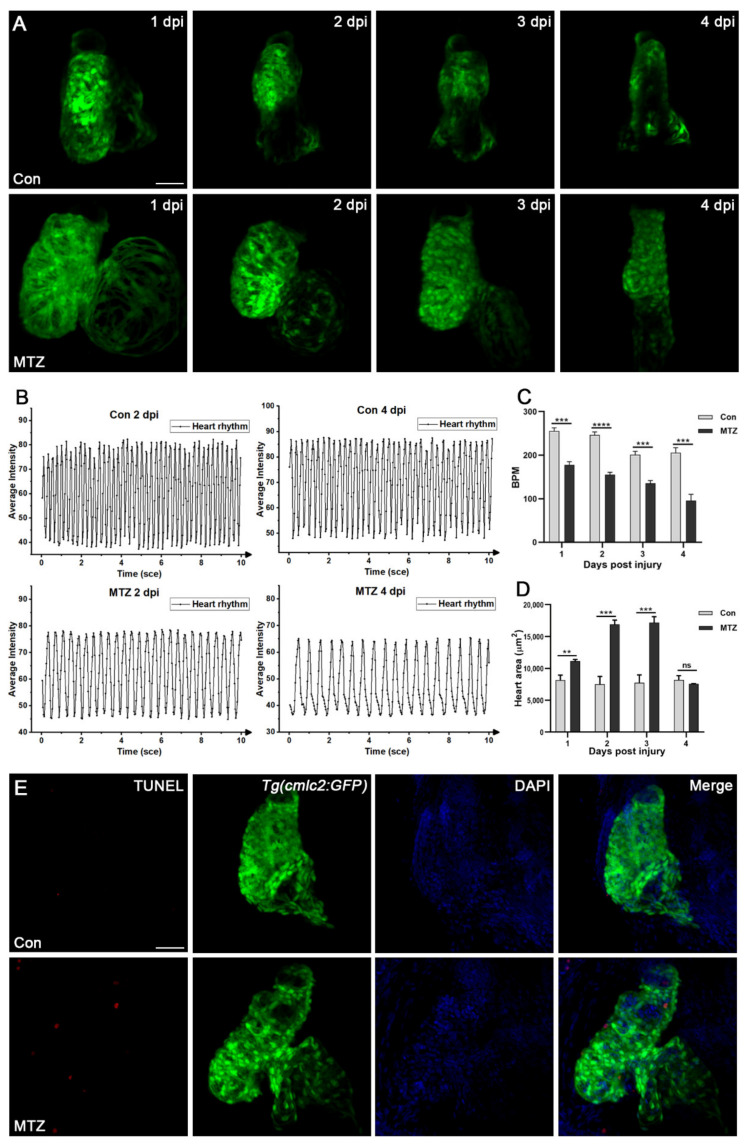
ARF induced heart failure in zebrafish larvae. The heart rate (**A**–**C**) and heart size (**D**) of *Tg(cdh17:Dendra2-NTR;cmlc2:GFP)* zebrafish larvae treated with MTZ or control were detected (*n* = 5). (**E**) TUNEL-positive apoptotic cardiomyocytes (red) were detected in the heart of *Tg(cdh17:Dendra2-NTR;cmlc2:GFP)* zebrafish larvae treated with MTZ, but not in the control (*n* = 30). Scare bar: 100 μm. Mean ± SEM; ns, not significant; ** *p* < 0.01, *** *p* < 0.001, **** *p* < 0.0001, ANOVA/Dunnett’s test compared with control.

Cardiac complications occur commonly in ARF patients [20] and are the leading cause of death. To observe the cardiac complications of the zebrafish ARF model, *Tg(cdh17:Dendra2-NTR)* transgenic fish were crossed with the *Tg(cmlc2:GFP)* line, which label cardiomyocytes. We found the average beat per minute (BPM) of the control group fluctuated between 201 and 255, while the average BPM of ARF larvae gradually decreased from 1 dpi to 4 dpi (177, 155, 135 and 95) (Figure 4A–C; Video S1). The heart enlargement or heart deformation of ARF larvae began to appear at 1 dpi and 2 dpi, respectively. Compared with the control group, the fluorescence intensity of ARF larvae hearts was weaker, and the arrangement of cardiomyocytes was looser and more disorderly (Figure 4D; Video S1). Besides, cardiomyocyte apoptosis in ARF larvae was observed by TUNEL assay (Figure 4E). All these cardiac changes in ARF larvae are highly consistent with those of human ARF patients. Therefore, ARF larvae can be used as a CRS-3 animal model.

**Figure 5:**
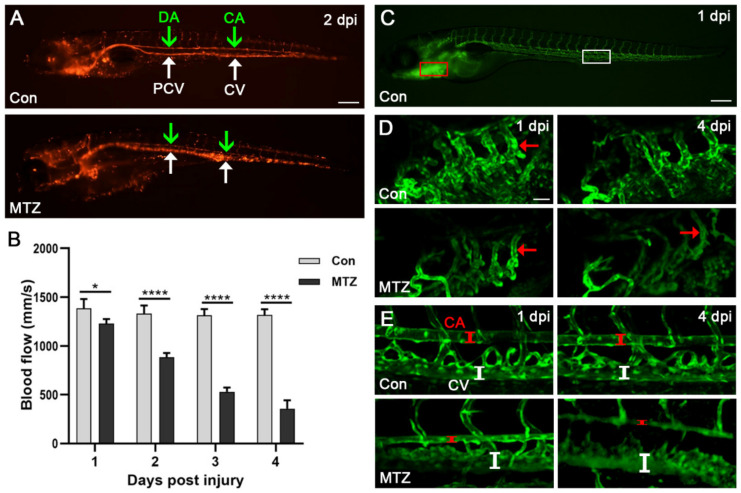
Changes of blood flow and blood vessels in ARF larvae. (**A**) The erythrocytes in the caudal artery (CA) (green arrow), caudal vein (CV) (white arrow), posterior cardinal vein (PCV) (green arrow) and dorsal aorta (DA) (white arrow) of *Tg(cdh17:Dendra2-NTR; flk1:GFP;gata1:DsRed)* zebrafish larvae treated with MTZ or control are shown. Scale bar: 200 μm. (**B**) After treatment with MTZ, the caudal artery blood flow velocity of zebrafish larvae (*n* = 5) was evaluated using ImageJ. (**C**–**E**) Confocal images of blood vessels around the heart and tail of *Tg(cdh17:Dendra2-NTR; flk1:GFP;gata1:DsRed)* zebrafish larvae treated with MTZ or control (*n* = 5). The Scale bars of (**C**) and (**D**,**E**) are 100 μm and 200 μm, respectively. The red and white line segments represent the vessel lumen of zebrafish larvae. CA, caudal artery; CV, caudal vein. Mean ± SEM; * *p* < 0.05 and **** *p* < 0.001, ANOVA/Dunnett’s test compared with control.

In addition, we crossed *Tg(cdh17:Dendra2-NTR)* with *Tg(flk1:GFP;gata1:DsRed)* transgenic fish, and fluorescently labeled blood vessels and erythrocytes. As shown in Figure 5A and Video S2, the video of erythrocytes’ movement reflected the changes in hemodynamics. We further found the blood flow velocity of the caudal artery in the control group was 1314–1384 mm/s (Figure 5B). After MTZ treatment, the blood flow velocity gradually decreased, from 1231 mm/s at 1 dpi to 357 mm/s at 4 dpi (Figure 5B). In addition, obvious caudal artery (CA) narrowing and caudal vein (CV) dilation were observed in ARF larvae at 4 dpi (Figure 5C–E). These apparent abnormalities regarding the heart, and vessels of ARF larvae are closely related to CRS-3 [20], which further shows that the zebrafish ARF model also can simulate human CRS-3.

### 3.5. Evaluation the Efficacy of Approved Therapeutic Drugs on the CRS-3 Model

**Figure 6:**
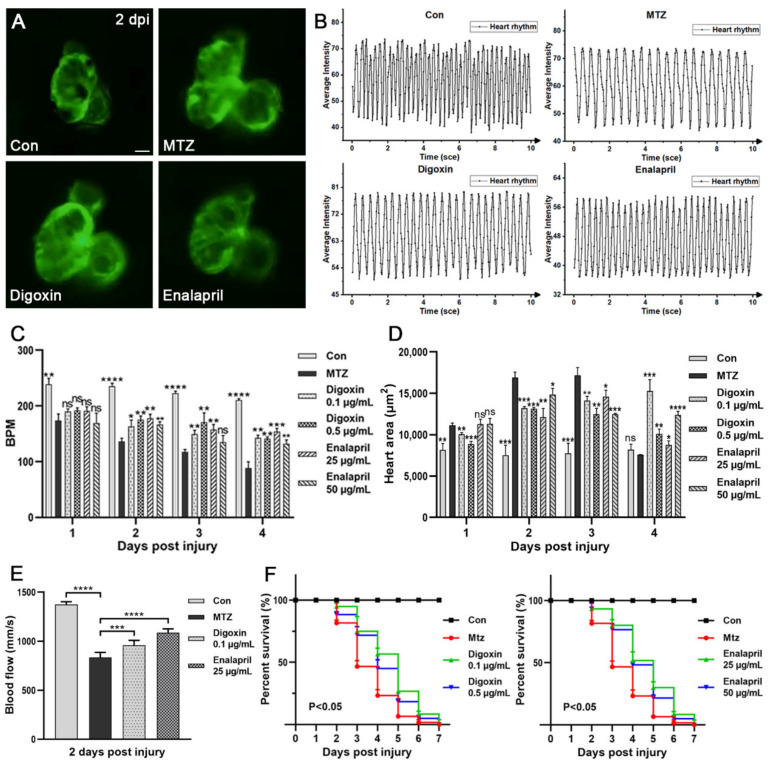
Evaluation of the effect of digoxin and enalapril on the CRS-3 model. (**A**) Heart images of zebrafish larvae (Control, zebrafish larvae treated with MTZ, zebrafish larvae treated with digoxin or enalapril) (*n* = 5) at 2 dpi are shown. Scale bar: 100 μm. (**B**,**C**) Heart rate was detected by the dynamic change pattern in the ventricle of zebrafish larvae. (**D**) ARF-induced heart enlargement can be decreased by digoxin and enalapril (*n* = 5). (**E**) Compared with the control group, treatment of ARF larvae with digoxin and enalapril resulted in significant increases in blood flow velocity (*n* = 5). (**F**) After treatment with digoxin or enalapril, the survival rate of ARF zebrafish larvae was increased (*n* = 60). Mean ± SEM; ns, not significant; * *p* < 0.05, ** *p* < 0.01, *** *p* < 0.001, and **** *p* < 0.0001, ANOVA/Dunnett’s test compared with untreated ARF zebrafish larvae.

In order to further verify the effectiveness of the zebrafish CRS-3 model, two FDA-approved heart failure drugs (digoxin and enalapril) were selected to treat ARF larvae, and the efficacy was evaluated by the heart, blood vessels and survival rate. Digoxin has beneficial hemodynamic and neurohormonal effects on patients with heart failure [21]. Enalapril can delay the clinical progression of congestive heart failure symptoms and reduce the need for hospitalization [22]. As shown in our results, compared with ARF larvae, after treatment with different concentrations of digoxin or enalapril, the symptoms of ARF zebrafish larvae with a heart-rate disorder appeared to have been significantly improved. At 1 dpi–4 dpi, the average BPM of ARF larvae treated with 0.1 μg/mL digoxin or 25 μg/mL enalapril were increased approximately 1.5 times (Figure 6A–C; Video S3). The symptom of heart enlargement was also improved after treatment with these therapeutic drugs (Figure 6D; Video S3). Furthermore, the blood flow velocity of ARF larvae was increased after treatment with digoxin or enalapril. At 2 dpi, the average caudal artery blood flow velocity of ARF larvae treated with 0.1 μg/mL digoxin or 25 μg/mL enalapril were 959 mm/s and 1088 mm/s, respectively (Figure 6E; Video S3). Both digoxin and enalapril treatment can enhance the survival rate of ARF larvae. As shown in Figure 6F, compared with the untreated group (81.6%, 46.67%, 23.33% and 6.667%), the average survival rates of ARF larvae treated with 0.1 μg/mL digoxin (95%, 75%, 56.67% and 26.67%) or 25 μg/mL enalapril (93.33%,80%, 51.67% and 30%) were significantly increased at 2 dpi to 5 dpi. This is consistent with the clinical efficacy of digoxin and enalapril in human clinical treatment, and also confirmed the applicability of this CRS-3 model in drug screening. However, due to the side effects of digoxin and enalapril, they can be effective only with the appropriate drug concentration and treatment regimens.

### 3.6. Screening Potential Drugs and Exploring Possible Mechanisms of Drugs Effect on CRS-3 Model

**Figure 7:**
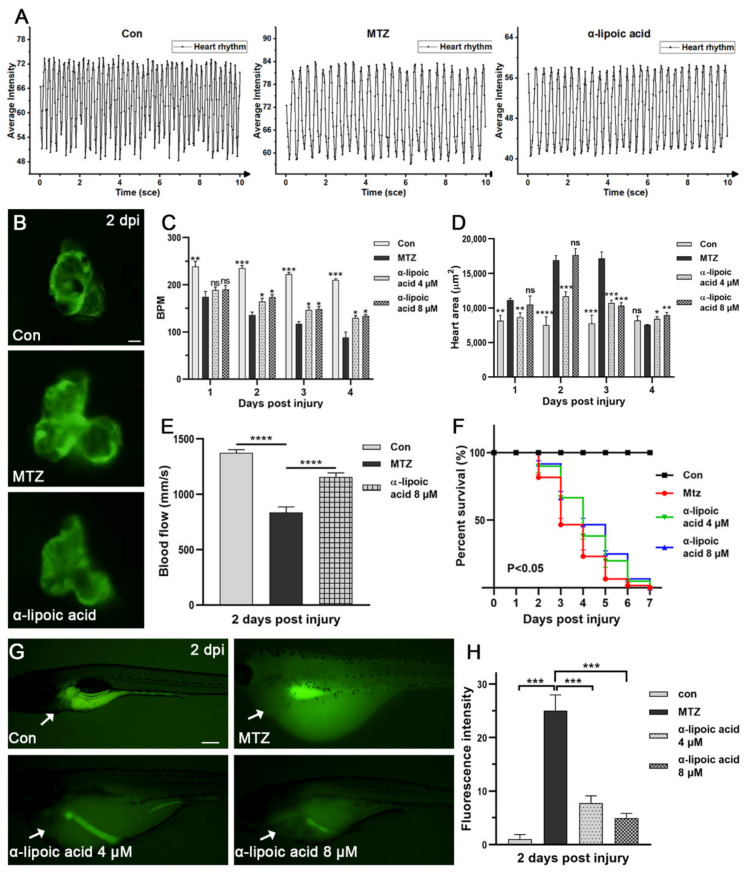
Effect of α-lipoic acid on CRS-3. (**A**–**F**) The heart rate, blood flow, heart dilatation and survival rate of ARF larvae treated with α-lipoic acid (4 μM, 8 μM) and the untreated group are presented. The number of larvae in each group was five, except for the survival rate group (*n* = 60). Scale bar: 100 μm. (**G**,**H**) MTZ-induced ROS in zebrafish larvae (*n* = 5) can be attenuated by α-lipoic acid. The image shows that ROS significantly decreased as the drug concentration increased. The white arrow points to the heart area. The Scale bar of (**B**,**G**) are 100 μm and 200 μm, respectively. Mean ± SEM; ns, not significant; * *p* < 0.05, ** *p* < 0.01, *** *p* < 0.001, and **** *p* < 0.0001, ANOVA/Dunnett’s test compared with untreated ARF larvae.

Next, in order to screen new CRS-3 therapeutic drugs, we tried several possible effective drugs (α-lipoic acid, curcumin, cinnamaldehyde) to treat ARF larvae. The effect of α-lipoic acid is the most obvious (Figure S3). After treatment of ARF larvae with α-lipoic acid, both the heart rate and blood flow velocity significantly increased, heart enlargement improved and the survival rate was significantly enhanced (Figure 7A–F; Video S4). Therefore, the effect of α-lipoic acid on the heart is similar to that of digoxin and enalapril. These results indicated that α-lipoic acid may be a new potential CRS-3 therapeutic drug.

As a powerful antioxidant, α-lipoic acid has significant electrophilicity and the ability to react with free radicals, and has a beneficial effect in clinical conditions [23,24]. Studies show that acute kidney injury leads to increased systemic oxidative stress, involving multiple pathological pathways of ARF, such as cardiovascular disease [25,26]. Therefore, we speculate that the antioxidant activity of α-lipoic acid may be involved in the therapeutic mechanism. We detected the changes of reactive oxygen species (ROS) in untreated or α-lipoic acid-treated ARF zebrafish larvae. We found that the ROS level of α-lipoic acid-treated ARF larvae was significantly decreased compared to the control and other drug treatment groups (Figure S3). The ROS level in the heart also decreased significantly (Figure 7G,H). These results support that the anti-ROS effect of α-lipoic acid is involved in the treatment mechanism of CRS-3, which also proved it has potential as a drug in CRS-3 treatment. Meanwhile, Digoxin and enalapril had no effect on the level of ROS, which proved that the causes of CRS-3 were diverse, and we can intervene in this disease through different targets.

## 4. Discussion

*cdh17* is a marker gene specifically expressed in renal tubular epithelial cells of zebrafish. Many transgenic lines have been constructed using the promoter of this gene, which can be used in the study of renal tubular development and damage repair [27,28,29]. However, there is no report on transgenic fish lines that can specifically damage renal tubular epithelial cells. Herein, we constructed a transgenic fish line of *Tg(cdh17:Dendra2-NTR)* that takes advantage of the MTZ/NTR system. When the transgenic larvae were treated with MTZ, most of the renal tubular epithelial cells could be ablated. This caused a sudden loss of renal function in zebrafish larvae. Therefore, we successfully constructed the zebrafish ARF model. This model can be used not only to study ARF, but also to study the effect of renal tubular epithelial cell ablation on other cell types.

Using the zebrafish ARF model, we found that zebrafish had cardiac complications that are highly similar to human CRS-3. To further verify the effectiveness of the zebrafish CRS-3 model, we tested the therapeutic effects of two FDA-approved heart failure drugs (digoxin and enalapril) on this CRS-3 model. After treatment, cardiac dilation and venous congestion of ARF larvae were significantly reduced. This indicates that the zebrafish CRS-3 model can not only simulate the symptoms of human patients, but also be used to screen and evaluate therapeutic drugs in vivo.

Zebrafish have now been used in large-scale drug screening for a variety of diseases. Using the ARF model, we further found that α-lipoic acid had a great effect on CRS-3. This proves that the zebrafish ARF model can be used for large-scale drug screening. Furthermore, we found that α-lipoic acid exerts its therapeutic function through the effect of antioxidant stress. This means that oxidative stress plays an important role in the pathogenesis of ARF complications. Next, we will use this model for large-scale drug screening, hoping to find new drugs with small side effects and great therapeutic effect for further clinical research.

However, the zebrafish ARF model inevitably has some defects. Because NTR is highly expressed in all renal tubular epitheliums, all renal tubular epithelial cells were injured by MTZ with the same intensity. This will cause extensive irreparable renal damage. This is different from many patients with AKI who still retain some renal function. Therefore, this model cannot be used to screen diuretics for ARF. In addition, due to the permeability of zebrafish larval skin [30,31], some water-soluble or small-molecular-weight urinary toxins, such as UN, may diffuse into the culture medium through the skin, which may be the reason the concentration of UN is lower than that of some other species’ ARF models [32,33,34]. However, from the phenotypes of ARF larvae, this permeability should not have a great impact on water and sodium retention or acid–base imbalance. The zebrafish ARF model also has the advantages of simple batch operation, low cost and high efficiency. The zebrafish is also a convenient model animal for genetic manipulation. Using CRISPR/Cas9 and other technologies, it is convenient to knock out specific genes in zebrafish, so as to study its role in the pathogenesis and treatment of ARF complications.

## 5. Conclusions

ARF often leads to numerous complications, among which CRS-3 is the main cause of death. In this study, the zebrafish ARF model was established, which can be used to screen and evaluate the therapeutic effect of CRS-3 drugs in vivo. We believe that this model is a convenient and efficient alternative to mammalian drug screening models. Furthermore, it will also have broad application in the research on the pathogenesis of ARF complications.

## Supplementary Materials

The following are available online at https://www.mdpi.com/article/10.3390/biom11091370/s1, Figure S1: Determination of MTZ treatment concentration; Figure S2: 8 mM MTZ does not cause zebrafish larvae nephrotoxicity; Figure S3: Effect of multiple drugs on ROS level of ARF larvae. Video S1. Changes in the heart of ARF larvae, Video S2. Changes in the blood flow of ARF larvae, Video S3. Effect of digoxin and enalapril on ARF zebrafish larvae, Video S4. Effect of α-lipoic acid on ARF zebrafish larvae.
