# Supplementary material for: Establishment of a Drug Screening Model for Cardiac Complications of Acute Renal Failure
Source: Biomolecules. 2021 Sep 16;11(9):1370. doi: 10.3390/biom11091370 (PMC8469377; doi:10.3390/biom11091370)
Supplement: Supplementary file 1 [file biomolecules-11-01370-s001.zip › Supplementary materials.pdf]

Supplementary Materials

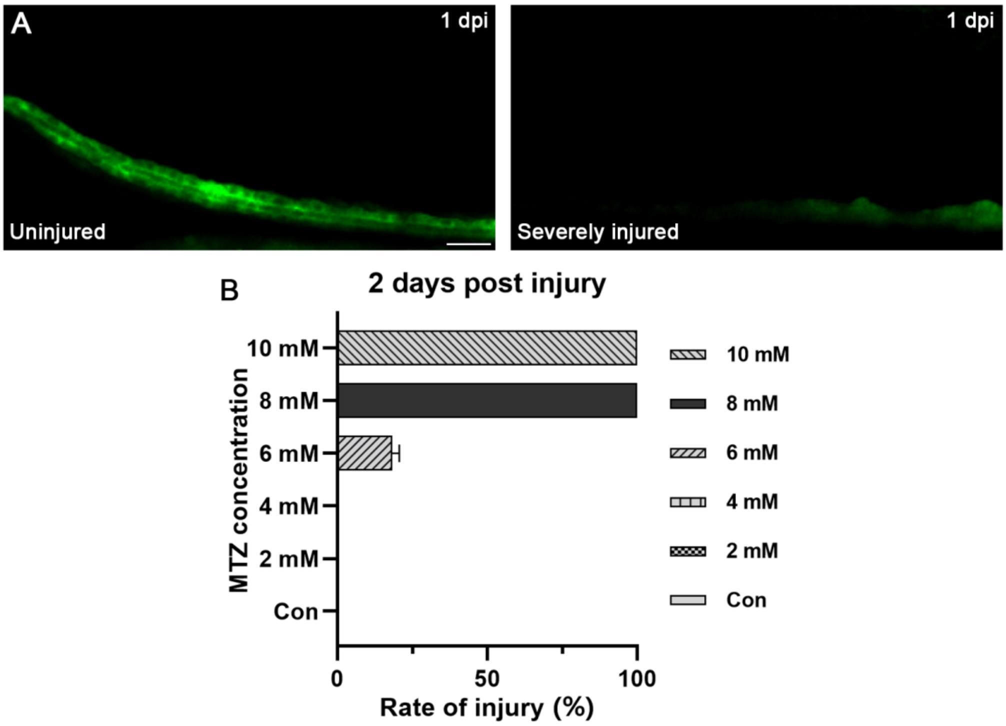

Figure S1. Determination of MTZ treatment concentration. Image of uninjured (A) and severely injured (B) renal tubules. (C) The renal tubule severely injured rate of *Tg(cdh17:Dendra2-NTR)* larvae was calculated upon exposure to MTZ (2 mM, 4 mM, 6 mM, 8 mM, 10 mM) 16h (n = 60). Scare bar: 100 μm; Mean ± SEM; ns, not significant; *p < 0.05, **p < 0.01, ***p < 0.001, and ****p < 0.0001, ANOVA/Dunnett's test compared with untreated ARF larvae.

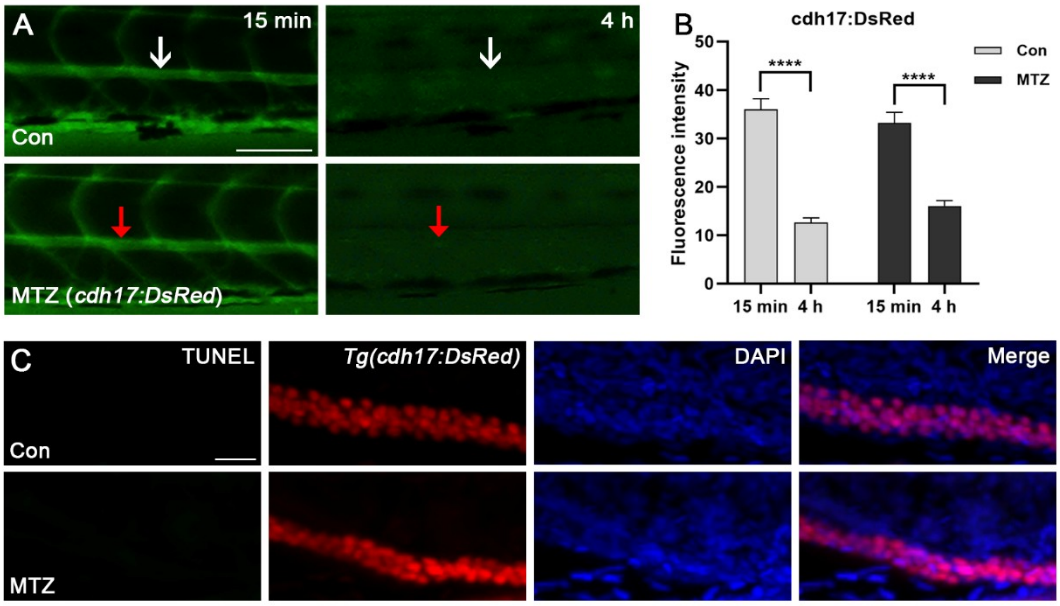

Figure S2. 8 mM MTZ does not cause zebrafish larvae nephrotoxicity. (A–B) *Tg(cdh17:DsRed)* and wild type zebrafish larvae were injected with 2 nl 5% FITC-inulin, and detected the FITC-inulin fluorescence intensity of the vascular network 15 minutes and 4 hours post-injection (n = 5). (C) TUNEL–positive apoptotic cardiomyocytes (red) were not detected in the heart of *Tg(cdh17:DsRed)* and wild–type zebrafish larvae (n = 30). Scare bar: 50 μm. Mean ± SEM; ns, not significant; *p < 0.05, **p < 0.01, ***p < 0.001, and ****p < 0.0001 (ANOVA/Dunnett's test).

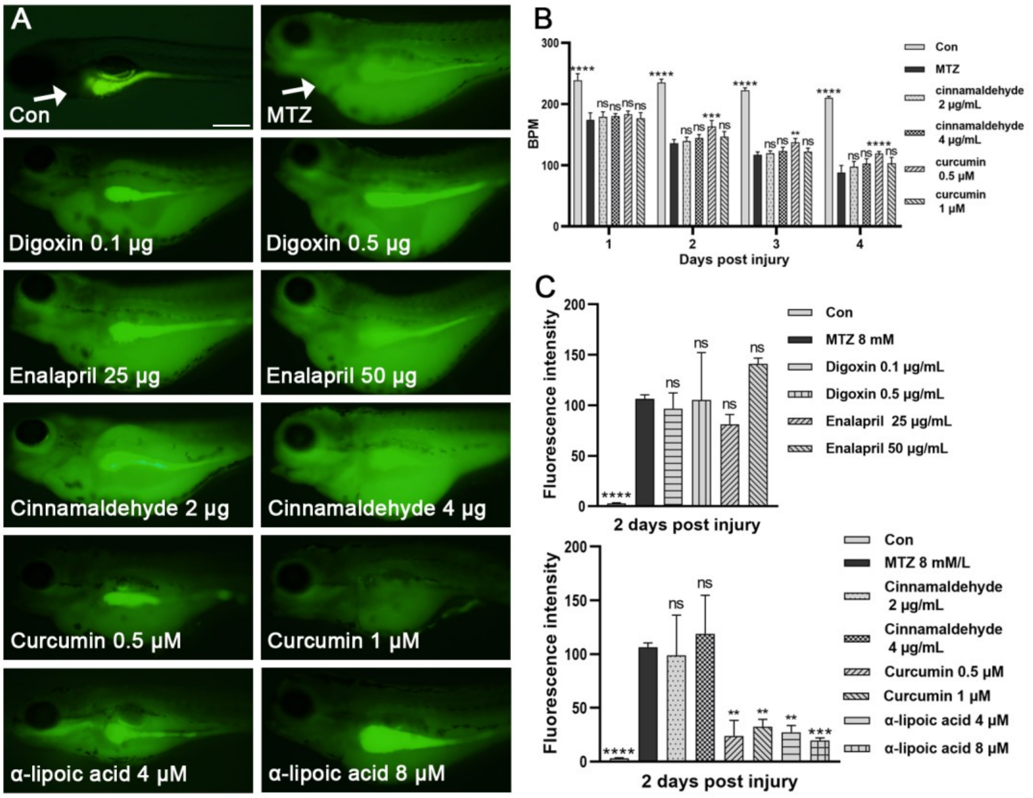

Figure S3. Effect of multiple drugs on ROS level of ARF larvae. (A) and (C) ROS of ARF larvae (n = 5) were detected at 2 dpi after treatment with digoxin (0.1 μg/mL and 0.5 μg/mL), enalapril (25 μg/mL, 50 μg/mL), cinnamaldehyde (2 μg/mL, 4 μg/mL), curcumin (0.5 μM, 1 μM), and α-lipoic acid (4 μM, 8 μM), respectively. (B) The heart rate of ARF larvae treated with cinnamaldehyde (2 μg/mL, 4 μg/mL) or curcumin (0.5 μM, 1 μM) were detected. Scare bar: 100 μm; Mean ± SEM; ns, not significant; *p < 0.05, **p < 0.01, ***p < 0.001, and ****p < 0.0001, ANOVA/Dunnett's test compared with untreated ARF larvae.

Video S1. Changes in the heart of ARF larvae. The dynamic heartbeat videos of zebrafish larvae treated with MTZ or control were captured with BX3-CBH microscope (Olympus, Japan). Compared to the control group, the video showed zebrafish larvae treated with MTZ occurred the symptoms of heart rate deceleration and heart enlargement at 2 dpi. The *Tg(cdh17:Dendra2-NTR;cmlc2:GFP)* transgenic fish was used. Imaging duration is 10 seconds. Related to Figure 4.

Video S2. Changes in blood flow of ARF larvae. The dynamic blood flow videos of ARF zebrafish larvae or control were captured with BX3-CBH microscope (Olympus, Japan). Videos displayed the blood flow velocity of zebrafish larvae treated with MTZ was reduced at 2 dpi compared with control. The *Tg(cdh17:Dendra2-NTR;flk1:GFP;gata1:DsRed)* transgenic fish was utilized. Video duration is 10 seconds. Related to Figure 5.

Video S3. Effect of digoxin and enalapril on ARF zebrafish larvae. Dynamic blood flow or heartbeat of ARF zebrafish larvae treated with drugs (digoxin, enalapril) or untreated group were captured with

BX3-CBH microscope (Olympus, Japan). Compared with the untreated group, videos shown that the symptoms of CRS-3, such as cardiac malformations, slowed heart rate, and reduced blood flow, were significantly improved. The *Tg(cdh17:Dendra2-NTR;cmlc2:GFP)* and *Tg(cdh17:Dendra2-NTR;flk1:GFP;gata1:DsRed)* transgenic fish were used. Video duration is 10 seconds. Related to Figure 6.

Video S4. Effect of α-lipoic acid on ARF zebrafish larvae. Using BX3-CBH microscope (Olympus, Japan) to capture dynamic blood flow or heartbeat of ARF zebrafish larvae treated with α-lipoic acid or untreated group. Videos displayed α-lipoic acid significantly improved the symptoms of CRS-3, such as slowed heart rate, cardiac deformation, and decreased blood flow compared with the untreated group. The *Tg(cdh17:Dendra2-NTR;cmlc2:GFP)* and Tg(cdh17:Dendra2-NTR;flk1:*GFP*;gata1:DsRed) transgenic fish were utilized. Video duration is 10 seconds. Related to Figure 7.
